# Heart failure and multimorbidity in Australian general practice

**DOI:** 10.15256/joc.2017.7.106

**Published:** 2017-04-28

**Authors:** Clare J. Taylor, Christopher Harrison, Helena Britt, Graeme Miller, FD Richard Hobbs

**Affiliations:** ^1^Nuffield Department of Primary Care Health Sciences, University of Oxford, Oxford, UK; ^2^Sydney School of Public Health, University of Sydney, Sydney, NSW, Australia

**Keywords:** heart failure, multimorbidity, aged, polypharmacy, general practice

## Abstract

**Background:**

Heart failure (HF) is a serious condition that mostly affects older people. Despite the ageing population experiencing an increased prevalence of many chronic conditions, current guidelines focus on isolated management of HF.

**Objective:**

To describe the burden of multimorbidity in patients with HF being managed in general practice in Australia.

**Design:**

Data from the Bettering the Evaluation And Care of Health (BEACH) programme were used to determine (i) the prevalence of HF, (ii) the number of co-existing long-term conditions, and (iii) the most common disease combinations in patients with HF. The study was undertaken over fifteen, 5-week recording periods between November 2012 and March 2016.

**Results:**

The dataset included a total of 25,790 general practitioner (GP) encounters with patients aged ≥45 years, collected by 1,445 GPs. HF had been diagnosed in 1,119 of these patients, a prevalence of 4.34% (95% confidence interval [CI] 3.99–4.68) among patients at GP encounters, and 2.08% (95% CI 1.87–2.29) when applied to the general Australian population overall. HF rarely occurred in isolation, with 99.1% of patients having at least one and 53.4% having six or more other chronic illnesses. The most common pair of comorbidities among active patients with HF was hypertension and osteoarthritis (43.4%).

**Conclusion:**

Overall, one in every 20–25 GP encounters with patients aged ≥45 years in Australia is with a patient with HF. Multimorbidity is a typical presentation among this patient group and guidelines for general practice must take this into account.

## Introduction

Heart failure (HF) is a clinical condition that is characterized by an inability of the heart to pump blood effectively around the body [1]. It is often the result of sustained pathological insult to cardiac tissues over a period of time and is, therefore, strongly associated with ageing [2]. Overall, HF affects around 1% of people in Western populations, but in older adults, the prevalence increases with each decade of life from 3% in those aged 65–74 years to 7% in those aged 75–84 years, and over 10% in those aged ≥85 years [3]. The average age at diagnosis of HF in the UK is 76 years [4]. Hospital admission is associated with up to 10% mortality [5]; however, patients diagnosed in the community have a better prognosis with almost one-third of patients surviving at least 10 years after diagnosis [6,7].

The current and projected burden of HF in Australia was recently reported by Chan *et al*. [8]. An estimated 480,000 Australians are currently living with HF at an annual cost of AUD 2.7 billion for community and in-patient care. The authors predict that demographic changes will result in the number of people with HF increasing to three-quarters of a million in the next 10–15 years, with an estimated annual healthcare cost of AUD 3.8 billion [8].

Multimorbidity is defined as the presence of two or more chronic diseases in an individual [9]. In a recent European survey of patients with HF in the community, 74% had at least one comorbid condition [10]. The workload for patients with multiple conditions is considerable; not only do they live with the burden of physical and/or mental illness, but they also have to manage multiple medications and medical appointments [11]. Healthcare systems need to recognize and be responsive to patient needs in order to enable optimal patient functioning [12].

As the population ages, healthcare providers need to deliver services capable of efficiently managing multimorbidity [13]. General practitioners (GPs) are well placed to manage multiple conditions, but there has been little research conducted to date on the conditions that commonly co-exist in patients with HF presenting to general practice. To allow clinical teams to provide individualized person-centred care, and to allow commissioners to plan healthcare services more broadly, the importance of quantifying the presence of multimorbidity in general practice is increasingly being recognized [14].

## Objective

The aim of this study was to quantify the burden of HF and multimorbidity in Australian general practice by determining (i) the prevalence of HF, (ii) the extent of multimorbidity among patients with HF, and (iii) the most common disease combinations.

## Materials and methods

### Setting and participants

This study used data from a sub-study of the BEACH programme. BEACH is a continuous, national cross-sectional study of general practice activity in Australia. The methods are described in detail elsewhere (BEACH 2015–16 annual report [15]). In brief, a random sample of approximately 1,000 GPs is selected each year to complete the BEACH project. By means of structured paper forms, each participating GP collects information about 100 consecutive encounters with consenting patients. In sub-studies of BEACH, the GP records additional information about the patient that may not necessarily relate to their encounter. Each GP undertakes three sub-studies during their 100 encounters (one 40 encounter sub-study and two 30 encounter sub-studies). In the current sub-study, 1,450 participating GPs were each asked to record the number of times the patient had attended general practice in the previous year and all diagnosed chronic conditions for each of the 30 consecutive patients within their 100 BEACH records. The study was undertaken over fifteen, 5-week recording periods between November 27, 2012 and March 28, 2016.

GPs were instructed to “Use your own knowledge, patient knowledge and health records as you see fit, in order to answer these questions”. GPs were first asked, “Approximately how many times has this patient seen *any* GP in the past 12 months (including today)?” They were then asked, “Does the patient have any chronic diseases/problems?” If the answer was ‘No’, the GP ended the questions for that particular patient. If the answer was ‘Yes’, the GP indicated all of the diagnosed chronic conditions for that patient; tick boxes were provided for common chronic conditions and additional blank spaces were provided to allow free text descriptions of other unlisted chronic conditions (See Supplementary Methods).

The chronic conditions that were listed (including HF) were primarily those that had been included in a previous prevalence study [16]; they were selected because they were the most frequent chronic conditions managed in Australian general practice. Chronic conditions were classified according to the International Classification of Primary Care (ICPC-2 Version 2) [17].

### Data analysis

We restricted our analysis to patients aged ≥45 years. Younger patients with HF have a different pathophysiology, which may have a genetic basis, and are also less likely to have co-existing diseases [18]. We first measured the proportion of patients with HF in the unweighted sample; this can be interpreted as the prevalence of that condition among patients at general practice encounters.

As patients were sampled at GP encounters, those who attended frequently (such as older patients who may have more health issues) were more likely to be sampled than those who attended infrequently. Therefore, patients at GP encounters are not representative of people in the general population. To account for this, we adjusted for high or low attenders by weighting each patient’s data by the number of times they saw a GP in the previous year, with high attenders being weighted down and low attenders being weighted up. The prevalence estimates created using this weighting refer to those who saw a GP at least once in the previous year, or “active patients”.

To provide estimates for the general population as a whole, we weighted the data further to match the age–sex distribution of the Australian population. We then adjusted each patient’s result by the proportion of people in their age–sex group (by 10-year age groups) who saw a GP at least once in that year (data supplied by the Australian Government Department of Health, personal communication). This adjusts for those patients who did not see a GP at least once in the previous year, as we assume they did not have diagnosed HF.

We estimated the prevalence of HF by age and sex for all patients aged ≥45 years. We then restricted the sample to those patients with HF and examined the prevalence of the twelve most common comorbidities, the prevalence of the number of comorbidities, and the most common comorbidity pairs.

BEACH sub-studies have a single-stage cluster design, with each GP having 30 patients clustered around them. Survey procedures (in SAS 9.3) were used to account for the effect of this clustering. Significant differences were determined by non-overlapping 95% confidence intervals (CIs). This is a more conservative estimate of difference than the usual probability <0.05 [19].

### Ethics statement

During the data collection period for this study, the BEACH programme was approved by the Human Research Ethics Committee of the University of Sydney (Reference number 2012/130).

## Results

### Heart failure prevalence

Data were collected from 1,445 GPs and included 25,790 GP encounters with patients aged ≥45 years. HF had been diagnosed in 1,119 of these patients, a prevalence of 4.34% (95% CI 3.99–4.68) among patients at GP encounters. After adjustment for patient attendance, an estimated 2.11% (95% CI 1.90–2.33) of active patients aged ≥45 years had diagnosed HF. After further adjustment (for age–sex distribution and GP attendance from Australian Government data, as described above), we estimated that 2.08% (95% CI 1.87–2.29) of people aged ≥45 years in the general Australian population had diagnosed HF.

The prevalence of HF by age and sex among sampled patients and in the general population are shown in Table 1. The prevalence of diagnosed HF in patients aged ≥45 years at GP encounters was significantly higher in males than in females (5.04% vs. 3.85%), especially among those aged 55–84 years. The prevalence of diagnosed HF at GP encounters increased significantly with age for both sexes. However, in the general population, no significant difference in the prevalence of diagnosed HF was found between the sexes in the overall group or subgroups by age. Nevertheless, the prevalence of HF increased significantly with age for both sexes.

### Heart failure and comorbidities

Nearly all patients with HF at GP encounters (99.1%) had at least one comorbid illness, with 90.6% having three or more, half (53.4%) having six or more, and 21.1% having nine or more comorbidities (i.e. HF plus nine or more additional diagnosed chronic conditions). The prevalence estimates for active patients with HF were very similar, with 98.2% having at least one comorbidity, 44.7% having six or more, and 16.9% having nine or more comorbidities.

Among sampled patients with HF aged ≥45 years, hypertension was the most common comorbidity, followed by osteoarthritis. Cardiovascular diseases such as atrial fibrillation and ischaemic heart disease were also common. However, unrelated diseases such as chronic back pain, depression and osteoporosis were present in more than one in five patients with HF (Table 2).

The average number of encounters that the active patients with HF had with a GP in the previous year was 12.4 (95% CI 11.6–13.1) (Table 2). Similar findings were observed when encounter rates were examined for active HF patients with each of the 10 most common comorbidities, with the exception of patients with HF and osteoporosis, who visited the GP significantly more often (14.8 [95% CI 13.1–16.6] visits) (Table 2).

The prevalence of the 10 most common pairs of comorbidities among active patients with HF aged ≥45 years is shown in Table 3. The most common pair of comorbidities was hypertension and osteoarthritis (43.4%), followed by hypertension and ischaemic heart disease (33.0%), and osteoarthritis and ischaemic heart disease (30.9%).

## Discussion

In Australia, HF is a common condition, with 4.34% (95% CI 3.99–4.68) of sampled patients aged ≥45 years at GP encounters having diagnosed HF. Multimorbidity was the norm amongst these patients with HF at GP encounters, with less than 1% having an isolated diagnosis of HF and just over half of them presenting with six or more comorbidities. In the active HF population, hypertension and osteoarthritis were the most common pairing of comorbidities. Cardiovascular comorbidities were also common and may share the same aetiology and also treatment strategies. Unrelated conditions such as depression and osteoporosis were also prevalent.

These figures clearly quantify the burden of HF in Australian general practice and could be useful for planning healthcare services. The study included a large number of GP encounters to maximize the accuracy of the prevalence estimates. Descriptive statistics of the number of comorbidities and the most common disease combinations are important to understand the scale of the problem. However, these measures alone are insufficient to explain the significance of each disease combination in terms of quality of life and survival. Further research is required to determine the relative influence of co-existing diseases on HF, and the combinations that are most burdensome and harmful.

The current findings are consistent with previous epidemiological studies [14,20]. Our estimated prevalence of diagnosed HF in the general population was 2.08% (95% CI 1.87–2.29), similar to the Echocardiographic Heart of England Screening (ECHOES) study, which reported a prevalence of 2.3% in the general population aged ≥45 years [20]. Barnett *et al*. [13] described the presence of multimorbidity in the Scottish population using medical records from over 1.75 million patients. Overall, multimorbidity was present in 64.9% (95% CI 64.7–65.1) of patients aged 65–84 years, and in 81.5% (95% CI 81.1–81.9) of those aged ≥85 years [13].

The challenge of multimorbidity in the context of HF as the index condition has not been previously well described. In our study, 99.1% of patients with HF aged ≥45 years had at least one comorbidity. This includes both physical and mental health conditions. One-quarter of participants with HF also had depression, which further complicates management and requires integration of care including community mental health services.

This study also demonstrates that HF is a disease of the elderly and does not exist in isolation [21,22]. There is some evidence that older patients with HF are less likely to receive guideline-recommended care [23]. However, most current HF guidelines globally are disease specific and the trials on which they are based often excluded patients with multimorbidity [24]. While the recent European Society of Cardiology guideline includes a chapter on comorbidities that commonly co-exist with HF, most patients have multimorbidity (two or more conditions) [1]. Optimizing the management of patients with multimorbidity is increasingly being recognized as an important area of research, and studies to find the best strategy are ongoing [25,26].

The findings of this study highlight to GPs and others the vital role of a generalist approach in managing patients with HF who frequently present with co-existing, often unrelated diseases. The management of these patients is often complex and requires time and input from a multidisciplinary team. Important drug–drug interactions can occur if single-disease guidelines are strictly followed [27]. Instead, a person-centred approach to establish issues of importance to the patient, provide holistic management of all their conditions, and avoid the adverse effects of polypharmacy are essential to optimize quality of care [28].

## Conclusion

Overall, one in every 20–25 GP encounters with patients aged ≥45 years in Australia is with a patient with diagnosed HF. Multimorbidity is widespread among this population, and guidelines must consider the implications for general practice. Importantly, the evidence base from single disease HF studies may not apply to most patients with multiple diseases who will be suffering an increased burden of illness and treatment. These patients warrant particular clinical attention and further research is required to determine the best approach.

## Figures and Tables

**Table 1:** Prevalence of heart failure (HF) in patients at general practitioner (GP) encounters and in the general population aged ≥45 years in Australia.

Age, years	Male HF prevalence, % (95% CI)			Female HF prevalence, % (95% CI)		
	*n*	Patients at GP encounters	General population	*n*	Patients at GP encounters	General population
45–54	2,129	0.70 (0.31–1.10)	0.17 (0.05–0.29)	3,348	0.24 (0.07–0.40)	0.10 (0.02–0.17)
55–64	2,607	1.99 (1.43–2.56)	0.88 (0.54–1.22)	3,582	0.75 (0.46–1.05)	0.48 (0.16–0.81)
65–74	2,757	4.10 (3.35–4.85)	2.25 (1.63–2.88)	3,593	2.64 (2.11–3.18)	1.80 (1.24–2.36)
75–84	2,114	9.22 (7.91–10.53)	6.08 (4.93–7.22)	2,947	6.79 (5.82–7.75)	4.94 (4.07–5.8)
≥85	897	17.17 (14.56–19.77)	11.98 (9.50–14.45)	1,658	15.26 (14.56–19.77)	11.35 (9.45–13.25)
≥45	10,504	5.04 (4.53–5.54)	2.21 (1.92–2.51)	15,128	3.85 (3.47–4.24)	1.98 (1.72–2.24)

CI, confidence interval.

**Table 2:** Prevalence of the 12 most common comorbidities and the number of general practitioner (GP) encounters in the previous year for active patients with heart failure (HF) in Australia.

Comorbidity	Prevalence, % (95% CI)		Number of GP encounters (95% CI)
	Patients at GP encounters	General population	
Active HF population	–	–	12.4 (11.6–13.1)
Hypertension	66.7 (63.7–69.7)	64.0 (59.9–68.1)	12.9 (11.9–13.8)
Osteoarthritis	65.1 (62.0–68.1)	59.7 (55.5–63.9)	13.5 (12.5–14.5)
Ischaemic heart disease	51.3 (47.9–54.7)	46.6 (42.4–50.8)	13.6 (12.7–14.6)
Hyperlipidaemia	38.9 (35.6–42.1)	38.4 (34.2–42.6)	12.5 (11.4–13.6)
Atrial fibrillation	37.7 (34.7–40.7)	33.7 (29.7–37.6)	13.9 (12.5–15.2)
Gastro-oesophageal reflux disease	28.5 (25.5–31.6)	24.8 (21.2–28.4)	14.2 (12.9–15.5)
Type 2 diabetes	28.2 (25.4–30.9)	25.9 (22.6–29.2)	13.4 (12.4–14.5)
Depression	25.6 (22.6–28.5)	21.5 (18.0–25.0)	14.7 (12.9–16.5)
Chronic obstructive pulmonary disease	22.8 (20.3–25.3)	20.2 (16.8–23.6)	14.0 (12.1–15.9)
Chronic back pain	21.9 (19.2–24.6)	18.7 (15.4–22.0)	14.5 (12.5–16.5)
Chronic renal failure	21.7 (18.8–24.6)	19.4 (15.8–23.0)	13.9 (12.0–15.8)
Osteoporosis	20.1 (17.5–22.7)	16.8 (13.9–19.6)	14.8 (13.1–16.6)

CI, confidence interval.

**Table 3:** Prevalence of the 10 most common pairs of comorbidities among active heart failure patients aged ≥45 years in Australia.

Pairs of comorbidities	Prevalence, % (95% confidence interval)
Hypertension and osteoarthritis	43.4 (39.3–47.4)
Hypertension and ischaemic heart disease	33.0 (29.4–39.6)
Osteoarthritis and ischaemic heart disease	30.9 (27.3–34.5)
Hypertension and hyperlipidaemia	29.8 (26.1–33.5)
Osteoarthritis and hyperlipidaemia	24.3 (20.9–27.6)
Hypertension and atrial fibrillation	23.8 (20.3–27.3)
Osteoarthritis and atrial fibrillation	22.6 (19.0–26.2)
Hyperlipidaemia and ischaemic heart disease	22.0 (18.8–25.2)
Hypertension and type 2 diabetes	19.8 (16.9–22.6)
Osteoarthritis and gastro-oesophageal reflux disease	17.6 (14.7–20.5)
